# Uridine stimulate laxative effect in the loperamide-induced constipation of SD rats through regulation of the mAChRs signaling pathway and mucin secretion

**DOI:** 10.1186/s12876-017-0576-y

**Published:** 2017-01-26

**Authors:** Ji Eun Kim, Jun Go, Ji Eun Sung, Hyun Ah Lee, Woo Bin Yun, Jin Tae Hong, Dae Youn Hwang

**Affiliations:** 1 Department of Biomaterials Science, College of Natural Resources & Life Science/Life and Industry Convergence Research Institute, Pusan National University, 50 Cheonghak-ri, Samnangjin-eup Miryang-si, Gyeongsangnam-do 627-706 Korea; 2 College of Pharmacy, Chungbuk National University, Chungju, 361-763 Korea

**Keywords:** Constipation, Uridine, Loperamide, Excretion parameters, Mucin, Muscarinic acetylcholine receptors

## Abstract

**Background:**

Uridine (Urd), which has been reported as a major component of RNA, plays an important role in various biological process including neuroprotection, biochemical modulation and glycolysis, although its role in constipation has yet to be established. Therefore, in this study, we investigated the laxative effects of Urd on chronic constipation.

**Methods:**

The constipation phenotypes and their related mechanisms were investigated in the transverse colons of SD rats with loperamide (Lop)-induced constipation after treatment with 100 mg/kg of Urd.

**Results:**

The number, weight and water contents of stools were significantly higher in the Lop + Urd treated group than the Lop + Vehicle treated group, while food intake and water consumption of the same group were maintained at a constant level. The thickness of the mucosa layer, muscle and flat luminal surface, as well as the number of goblet cells, paneth cells and lipid droplets were enhanced in the Lop + Urd treated group. Furthermore, the expression of the muscarinic acetylcholine receptors M2 and M3 (mAChR M2 and M3) at the transcriptional and translational level was recovered in the Lop + Urd treated group, while some markers such as Gα and inositol triphosphate (IP3) in their downstream signaling pathway were completely recovered by Urd treatment. Moreover, the ability for mucin secretion and the expression of membrane water channel (aquaporine 8, AQP8) were increased significantly in the Lop + Urd treated group compared with Lop + Vehicle treated group. Finally, the activity of Urd was confirmed in primary smooth muscle of rat intestine cells (pRISMC) based on Gα expression and IP3 concentration.

**Conclusions:**

The results of the present study provide the first strong evidence that Urd can be considered an important candidate for improving chronic constipation induced by Lop treatment in animal models.

## Background

Constipation is an acute or chronic gastrointestinal disease characterized by infrequent bowel movements, hard and dry feces, incomplete bowel evacuation and difficulty during defecation [1]. Although a variety of treatments for this disease are known, the best method for treatment of constipation is making simple changes to incorporate more fiber into the diet, drink plenty of fluids, and add exercise into the patient’s daily routine. In most cases, chemical drugs (laxatives) such as senna, correctol, exlax, senokot and gaviscon can be widely prescribed to help patients pass stools [2]. These drugs act as stimulants to increase bulkiness and soften stool, or as osmotic agents, enhancing water flow into the colon to promote elimination and trigger bowel movements. However, most of these agents have undesirable side effects including artery contraction, coronary spasms and myocardial infarction [3–5]. Therefore, identification of novel laxatives with no side effects has been the main focus of the constipation treatments.

Urd, which is one of the five standard nucleosides that makes up nucleic acids, is a glycosylated pyrimidine-analog consisting of uracil linked to a ribose ring [6]. After phosphorylation to nucleotides, Urd was used for the synthesis of nucleic acid and membrane constituents, as well as glycosylation [7]. Moreover, Urd is widely involved in a variety of biochemical processes including the glycolysis pathway, biochemical modulation and neuroprotection. Urd plays a role in the glycolysis pathway of galactose even if catabolic processes for the metabolism of galactose are not involved this process [8]. Furthermore, Urd has been used successfully as a biochemical modulating agent to alleviate the side effects of various anti-cancer and anti-HIV drugs, although fever and diarrhea have been considered the major factors limiting increasing the dose of Urd [9–11]. Urd is the major form of pyrimidine nucleoside taken up by the brain, and it has been suggested that it be used as a neuroprotective agent to treat epileptic and neurodegenerative diseases, including depression and Alzheimer’s disease [7, 12]. Urd acts as a central nervous system (CNS) depressant, having anticonvulsant effects and inducing decreased spontaneous activity of adult male C-57 mice [13, 14] Although studies conducted to date have provided some information regarding the possibility of a correlation between intestinal bowel disease and Urd, no studies have investigated the laxative effects of Urd in the constipated animal model.

Therefore, the present study was conducted to verify the laxative activity and action mechanism of Urd in a Lop-induced constipation model. Our results provide the first scientific evidence that Urd can successfully induce laxative effects in the constipated animal model without any significant side effects.

## Methods

### Experimental design for animal study

Adult SD rats purchased from Samtako Inc. (Osan, Korea) were handled in the Pusan National University-Laboratory Animal Resources Center, which is accredited by the Korea Food and Drug Administration (FDA)(Accredited Unit Number-000231) and AAALAC International according to the National Institutes of Health guidelines (Accredited Unit Number; 001525). Animals were provided with *ad libitum* access to a standard irradiated chow diet (Samtako Inc.) and water. During the experiment, rats were maintained in a specific pathogen-free (SPF) state under a strict light cycle (lights on at 08:00 h and off at 20:00 h) at 23 ± 2 °C and 50 ± 10% relative humidity.

Constipation of SD rats was induced by subcutaneously injection of Lop as previously described [5, 15]. First, 8-week-old SD rats (*n* = 28) were assigned to either a non-constipation group (No group, *n* = 14) or a constipation group (*n* = 14). Constipation was induced by subcutaneous injection of Lop (4 mg/kg weight) in 0.5% Tween 20 in saline twice a day for 3 days, whereas the non-constipation group was injected with 0.5% Tween 20 in saline alone. The non-constipation group was further divided into a No treated group (*n* = 7) and a Urd treated group (*n* = 7). The No treated group was untreated during the experimental period, whereas the Urd treated group received 100 mg/kg Urd (Sigma-Aldrich Co., Saint Louis, MO, USA) at one time. Additionally, the constipation group was further divided into a Lop + Vehicle treated group (*n* = 7) and Lop + Urd treated group (*n* = 7). The Lop + Urd treated groups were orally administered 100 mg/kg body weight Urd at once, while the Lop + Vehicle treated group received the same volume of 1× PBS under the same pattern. At 24 h after Urd treatment, all SD rats were euthanized using CO_2_ gas and tissue samples were acquired and stored in Eppendorf tubes at −70 °C until assay.

### Analysis of food intake, water intake and body weight

Alterations in food intake, water consumption and body weight of SD rats treated with Lop + Urd were measured daily at 10:00 am throughout the experimental period using an electrical balance and a measuring cylinder. All measurements were performed three times to ensure accuracy.

### Measurement of stool parameters

During all experimental periods, SD rats of all groups were bred in metabolic cages to harvest pure stools and urine without any contamination (Daejong Instrument Industry Co., LTD, Seoul, Korea). The stool number and weight were measured as previously described [15, 16]. The stools excreted from each SD rat were collected at 10:00 am. Stool samples were weighed three times per sample using an electric balance, whereas the number of stools was counted three times.

### Western blotting

Total proteins were extracted from the transverse colons of subset groups (No, Urd, Lop + Vehicle and Lop + Urd treated SD rats) and pRISMCs using Pro-Prep Protein Extraction Solution (Intron Biotechnology, Inc., Seongnam, Korea). Following centrifugation at 13,000 rpm and 4 °C for 5 min, the protein concentrations were determined using a SMARTTM Bicinchoninic Acid Protein assay kit (Thermo Fisher Scientific, Inc.). Proteins (30 μg) were separated by 4–20% sodium dodecyl sulfate-polyacrylamide gel electrophoresis (SDS-PAGE) for 3 h, after which the resolved proteins were transferred to nitrocellulose membranes for 2 h at 40 V. Each membrane was then incubated separately with primary antibody, anti-mAChR M2 antibody (Alomone Labs, Jerusalem, Israel), anti-PI-3K (Cell Signaling Technology Inc., Cambridge, MA, USA), anti-p-PI3K (Cell Signaling Technology Inc.), anti-mAChR M3 antibody (Alomone Labs, Jerusalem, Israel), anti-PKC (Cell Signaling Technology Inc.), anti-p-PKC (Cell Signaling Technology Inc.), anti-Gα (Abcame, Cambridge, UK) or anti-actin (Sigma-Aldrich Co.) overnight at 4 °C. Next, the membranes were washed with washing buffer (137 mM NaCl, 2.7 mM KCl, 10 mM Na_2_HPO_4_, 2 mM KH_2_PO_4_, and 0.05% Tween 20) and incubated with horseradish peroxidase-conjugated goat anti-rabbit IgG (Zymed Laboratories, South San Francisco, CA, USA) at a dilution of 1:1000 and room temperature for 2 h. Finally, the membrane blots were developed using Chemiluminescence Reagent Plus kits (Pfizer, New York, NY, USA and Pharmacia, New York, NY, USA).

### RT-PCR

Total RNA was isolated from the frozen tissue of transverse colons using RNAzol B solution (Tet-Test Inc.) according to manufacturer’s protocols. After the synthesis of cDNA, genes were amplified by subjecting the samples to 28 cycles of 30 s at 94 °C, 30 s at 62 °C and 45 s at 72 °C in a Perkin-Elmer Thermal Cycler. The primer sequences used to evaluate mAChR M2 expression were as follows: sense primer, 5′-CCAGT ATCTC CAAGT CTGGT GCAAG G-3′, antisense primer, 5′-GTTCT TGTAA CACAT GAGGA GGTGC-3′. The primer sequences used to evaluate mAChR M3 expression were as follows: sense primer, 5′-GTCAC TTCTG GTTCA CCACC AAGAG C-3′, antisense primer, 5′-GTGTT CACCA GGACC ATGAT GTTGT AGG-3′. The primer sequences used to evaluate AQP8 expression were as follows: sense primer, 5′-GTAGT ATGGA CCTAC GTGAG ATCAA GG-3′, antisense primer, 5′-AGAAC CTTTC CTCTG GACTC ACCAC C-3′. The primer sequences used to evaluate MUC2 expression were as follows: sense primer, 5′-GCTGC TCATT GAGAA GAACG ATGC-3′, antisense primer, 5′-CTCTC CAGGT ACACC ATGTT ACCAG G-3′. The sequences of the β-actin sense and antisense primers were 5′-TGGAA TCCTG TGGCA TCCAT GAAAC-3′ and 5′-TAAAA CGCAG CTCAG TAACA GTCCG-3′, respectively. The PCR products were quantified using 1% agarose gels and a Kodak Electrophoresis Documentation and Analysis System 120.

### Histopathological analysis

Transverse colons collected from No, Urd, Lop + Vehicle and Lop + Urd treated SD rats were fixed with 10% formalin for 48 h, embedded in paraffin wax, and then sectioned into 5 μm thick slices that were stained with hematoxylin and eosin (H&E, Sigma-Aldrich Co.). Morphological features of these sections were observed by light microscopy, after which the mucosa thickness, muscle thickness, flat luminal surface thickness, number of goblet cells and number of lipid droplets were measured using Leica Application Suite (Leica Microsystems, Switzerland).

For mucin staining, transverse colons collected from SD rats that had been cotreated with Lop + Urd were fixed with 10% formalin for 48 h, embedded in paraffin wax, and then sectioned into 4 μm thick slices that were subsequently deparaffinized with xylene and rehydrated. Next, the tissue sections on the slides were rinsed with distilled water and stained with an Alcian Blue Stain kit (IHC WORLD, Woodstock, MD, USA). Finally, the morphological features in the stained colon sections were observed by light microscopy.

### Ultrastructure analysis using transmission electron microscopy (TEM)

The transverse colons collected from SD rats of subset groups were fixed in 2.5% glutaraldehyde in 1× PBS buffer, washed, dehydrated with ascending concentrations of ethanol, incubated in 1% OsO_4_ for 1 h at room temperature, and then embedded in Epon812 media (Polysciences Inc., Eppelheim, Germany). Ultra-thin sections (70 nm) were subsequently collected on holey formvar coated grids, contrasted with uranyl acetate and lead citrate, and examined by TEM (Hitachi, Tokyo, Japan) at 4000× magnification.

### Measurement of IP3 concentration

The levels of IP3 were determined using an IP3 ELISA kit (Cusabio Biotech Co., Ltd., Wuhan, China) according to the manufacturer’s instructions. Briefly, the pRISMCs (5 × 10^7^) were washed and homogenized in ice-cold PBS (pH 7.2–7.4) with a glass homogenizer (Sigma-Aldrich Co.). The cell lysates were then centrifuged at 1000 rpm for 5 min at room temperature, after which the supernatant was collected for analysis. An anti-IP3 detection antibody was added and incubated at 37 °C for 60 min, after which substrate solution was added and the samples were incubated for 15 min at 37 °C. The reaction was terminated following the addition of stop solution and the plates were read at an absorbance of 450 nm using a Molecular Devices VERSA max Plate reader (Molecular Devices, Sunnyvale, CA, USA).

### Treatment and preparation of pRISMC

pRISMCs used in this study were prepared from intestines of infant rats (3 days old)(*n* = 3) as previously described, with some modification [17]. Moreover, the purity of the pRISMCs population was confirmed by RT-PCR analysis with some modification as previously described [18]. Amplification was conducted in a Perkin-Elmer Thermal Cycler using the following cycles: 30 s at 94 °C, 30 s at 62 °C, and 45 s at 72 °C. The primer sequences for target gene expression identification were as follows: Myh11 (myosin-smooth muscle cells marker), sense primer: 5′-GCAAC TGAGC AATGA GCTGG TCAC-3′, anti-sense primer: 5′-CTGCT CCTTG TACTG CTCCA CCATC-3′; PGP9.5 (neuronal cell marker), sense primer: 5′-TACTT CATGA AGCAG ACCAT CG-3′, anti-sense primer: 5′-CTGCA GCAGA GAGTC CTCTG AACTG-3′; β-actin, sense primers; 5′-TGG AAT CCT GTG GCA TCC ATG AAA C-3′, anti-sense primer: 5′-TAA AAC GCA GCT CAG TAA CAG TCC G-3′. The final PCR products were separated on 1.2% agarose gel and then visualized by ethidium bromide staining. Among the two markers, only Myh11, indicating myosin-smooth muscle cells, was detected at high levels, while PGP9.5, indicating neuronal cells, was found at an extremely low level (Fig. 6a).

To treat Urd, pRISMCs were seeded at a density of 1 × 10^7^ cells/10 ml in 100 mm-diameter culture dishes, then grown with 20 μM Lop in a 37 °C incubator. After 12 h, cells were removed from the culture media with Lop and incubated with 100 μM Urd or 1× PBS for another 12 h. Next, cells harvested from 100 mm diameter culture dishes were used to determine the IP3 concentration and for western blot analysis.

### Statistical analysis

One-way ANOVA (SPSS for Windows, Release 10.10, Standard Version, Chicago, IL, USA) was used to determine the variance and identify significant differences between the No treated group and others groups, as well as between the Vehicle treated group and Urd treated group within the constipation group. All values are presented as the means ± standard deviation (SD). A *p* <0.05 was considered significant.

## Results

### Effect of Urd administration on feeding behavior and excretion parameters

To investigate whether Urd administration could affect the feeding behavior and excretion parameters of constipated rats, alterations in food intake, water consumption, stool number, and weight and water contents of stools were measured in Lop-induced constipated SD rats after Urd administration. As shown Table 1, no significant alterations in body weight, food intake or water consumption were observed. However, the decreases in the number, weight and water contents of stools in the Lop + Vehicle treated group were almost recovered in the Lop + Urd treated groups relative to those in the No and Urd treated group, although there were a few differences in the recovery rate. The opposite pattern was observed for the urine volume (Table 1).

**Table 1:** Measurement of body weight, feeding behavior, stools and urine secretion in Lop-induced constipated SD rats

Contents	No	Urd	Lop	
			Vehicle	Urd
Body weight (g)	270 ± 11.1	267 ± 14.5	261 ± 18.7	254 ± 17.4
Feeding behavior				
Food intake (g/day)	18.3 ± 2.2	18.4 ± 1.5	16.4 ± 1.9	16.1 ± 2.0
Water consumption (ml/day)	14.3 ± 2.1	15.7 ± 2.1	13.3 ± 4.1	15.7 ± 1.9
Stool				
Stool number (ea)	64.7 ± 9.5	65.7 ± 9.7	38.0 ± 13.1*	67.5 ± 12.2^#^
Stool weight (g)	8.8 ± 1.0	9.0 ± 1.5	5.2 ± 2.2*	10.0 ± 2.2^#^
Water content (%)	35.3 ± 3.1	35.9 ± 3.2	14.6 ± 0.8*	30.8 ± 3.4^#^
Urine volume (ml/day)	13 ± 1.6	13.3 ± 1.2	20.7 ± 1.7*	15.3 ± 1.7^#^

Data represent the means ± SD from three replicates. *p < 0.05 compared to the No treated group. ^#^ *p* < 0.05 compared to the Lop + Vehicle treated group. Urd; uridine, Lop; loperamide

### Effect of Urd administration on histological structure of the transverse colon

**Fig. 1:**
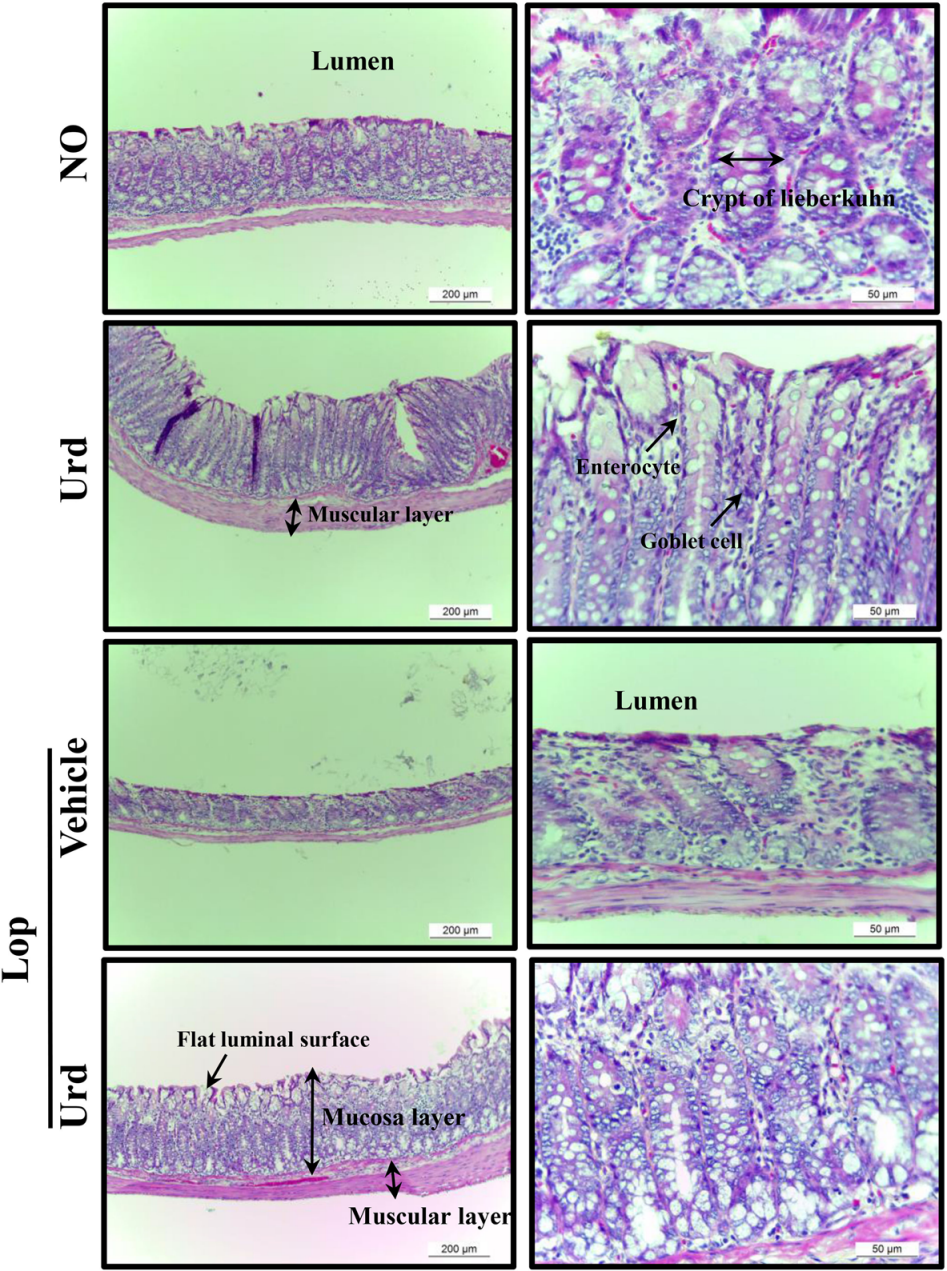
Alteration of histological structures in Lop-induced constipated rats. H&E stained sections of transverse colon rats from the No, Urd, Lop + Vehicle or Lop + Urd treated group were observed at 100× (left column) and 200× (right column) using a light microscope. Five to six rats per group were assayed in triplicate by H&E

We investigated whether Urd treatment could induce alterations in the histological structure of the transverse colon. To accomplish this, alterations in the histological parameters indicating laxative effects were measured in the H&E stained transverse colons of subset groups. The Urd treated group showed a similar structure as the No treated group. A significant decrease in the thickness of mucosa, muscle and flat luminal surface was observed in the Lop + Vehicle treated group relative to the No treated group and the Urd treated group. However, these levels dramatically increased by 283, 219 and 39% following Lop + Urd cotreatment when compared with the Lop + Vehicle treated group (Fig. 1 and Table 2). Furthermore, the number of goblet cells, crypt of lieberkuhn and enterocytes were 41, 54 and 34% lower in the Lop + vehicle treated group than the No treated group, respectively (Table 2). However, these levels were recovered in the Lop + Urd treated groups, although not completely to those of the No treated group (Fig. 1 and Table 2).

**Table 2:** Histopathological alterations in constipated SD rats after Urd treatment

Contents	No	Urd	Lop	
			Vehicle	Urd
Mucosa layer thickness (μm)	394 ± 35.2	415 ± 21.3	105 ± 12.5*	402 ± 23.5^#^
Muscle thickness (μm)	89.3 ± 6.5	92.3 ± 7.6	28.3 ± 4.3*	90.2 ± 6.6^#^
Flat luminal surface thickness (μm)	16.1 ± 3.2	15.4 ± 2.3	10.6 ± 1.1*	14.8 ± 2.0^#^
Number of goblet cells (ea)	85.6 ± 8.5	88.4 ± 6.7	51.2 ± 5.5*	72.6 ± 9.8^#^
Number of crypt of lieberkuhn (ea)	12.5 ± 2.6	14.2 ± 1.5	5.8 ± 1.1*	11.2 ± 2.1^#^
Number of enterocytes (ea)	125.2 ± 8.6	117.8 ± 7.7	82.3 ± 9.2*	102.4 ± 7.8^#^

*p < 0.05 compared with the non-constipation group. ^#^ *p* < 0.05 compared with the Lop + Vehicle treated constipation group. Urd; uridine, Lop; loperamide

### Effect of Urd administration on ultrastructure of the transverse colon

**Fig. 2:**
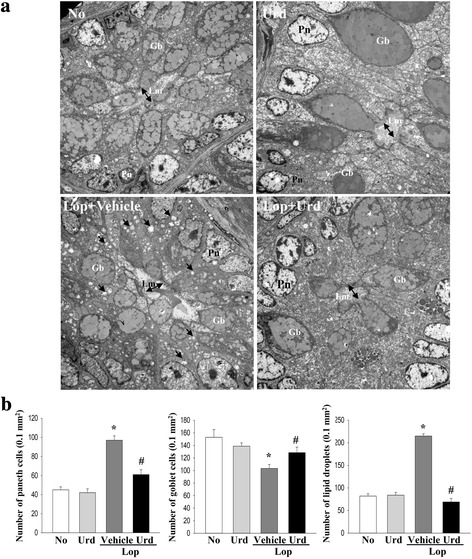
Ultrastructure image of transverse colon. **a** The ultrastructure of the crypt in the No, Urd-, Lop + Vehicle and Lop + Urd treated group was viewed by TEM at 4000× magnification. **b** The number of paneth cells, lipid droplets and goblet cells was measured using Leica Application Suite (Leica Microsystems, Switzerland). Five to six rats per group were assayed in triplicate by TEM analysis. The arrow indicates a lipid droplet distributed around the lumen of the crypt. Data represent the mean ± SD from three replicates. *, *p* < 0.05 compared to the No treated group. ^#^, *p* < 0.05 compared to the Lop + Vehicle treated group. Lm, lumen of crypt; Gb, goblet cells; Pn, paneth cells

Based on the above histopathological alterations, we examined whether Urd administration could accompany alterations in the ultrastructure of the transverse colon. To accomplish this, ultrastructural changes were observed in thin tissue section of the transverse colon using TEM analysis. In the No treated group and the Urd treated group, the Crypt of Lieberkuhn was clearly observed as a ring structure in which enterocytes, goblet cells, and paneth cells surrounded a lumen at the center. Following Lop treatment, the ultrastructure of the crypt changed dramatically. The number of paneth cells and lipid droplets was also higher in the Lop + Vehicle treated group than in the No-treated group. However, their levels were significantly recovered or they disappeared from around the cryp lumen in the Lop + Urd treated group. The decrease in the average size and number of goblet cells in the Lop + Vehicle treated group was also enhanced in the Lop + Urd treated group (Fig. 2).

### Correlation between the laxative effects of Urd and downstream signaling pathway of mAchRs

**Fig. 3:**
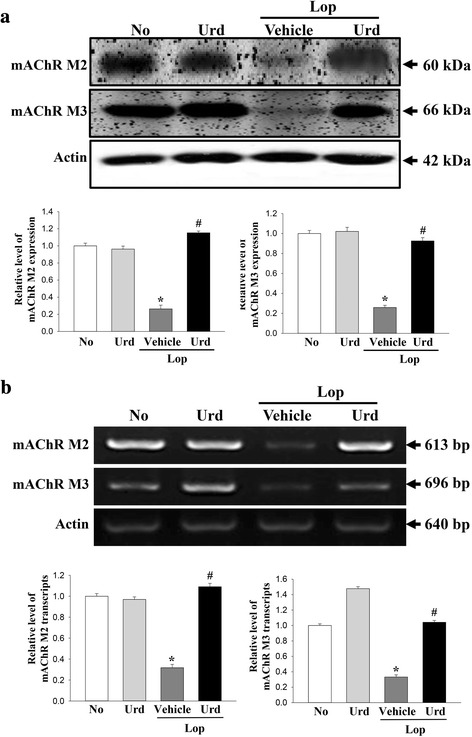
Expression of mAChRs transcript and protein in the transverse colon. **a** The expression of mAChR M2 and M3 proteins was measured by Western blot analysis using HRP-labeled anti-rabbit IgG antibody. **b** The levels of mAChR M2 and M3 transcripts in the total mRNA of transverse colons were measured by RT-PCR using specific primers. After the intensity of each band was determined using an imaging densitometer, the relative levels of mAChR M2 and M3 were calculated based on the intensity of actin. Five to six rats per group were assayed in triplicate by western blot and RT-PCR assays. Data represent the means ± SD of three replicates. *, *p* < 0.05 compared to the No treated group. ^#^, *p* < 0.05 compared to the Lop + Vehicle treated group

We next investigated the molecular mechanism of the laxative effects of Urd in the constipation model. To accomplish this, the mRNA and protein of mAChRs M2 and M3 were measured in the transverse colon of the Lop + Urd treated group using specific primers and antibodies. A similar alteration pattern in the mRNA and proteins of mAChRs M2 and M3 was observed in the subset group. The levels of these two receptors were lower in the Lop + Vehicle treated group than the No treated group and the Urd treated group. However, their levels rapidly increased by 303 and 395% in the Lop + Urd treated group (Fig. 3).

**Fig. 4:**
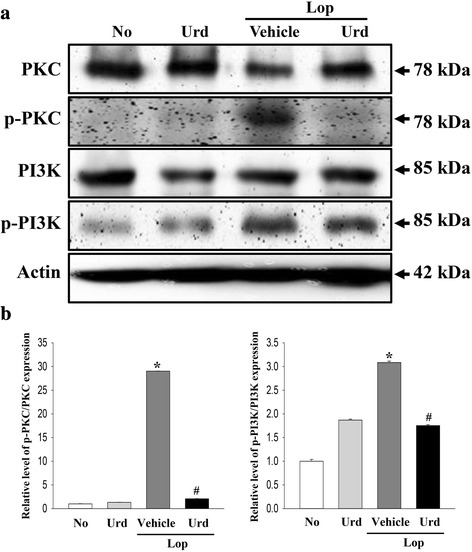
Expression of key proteins in the mAChR M2 and M3 downstream signaling pathway. The expression of several related proteins including PKC, p-PKC, PI3K and p-PI3K was measured by Western blot analysis using HRP-labeled anti-rabbit IgG antibody. After the intensity of each band was determined using an imaging densitometer, the relative levels of four proteins were calculated based on the intensity of actin protein. Five to six rats per group were assayed in triplicate by Western blotting. Data represent the means ± SD of three replicates. *, *p* < 0.05 compared to the No treated group. ^#^, *p* < 0.05 compared to the Lop + Vehicle treated group

Significant alterations in the expression of downstream of mAChR were also observed. The overall change in all experimental groups was very similar to the changes in the expression of PKC, p-PKC, PI3K and p-PI3K proteins. Specifically, these levels were dramatically increased by 2900 and 300%, respectively, in the Lop + Vehicle treated group relative to the No and Urd treated group. However, their levels significantly recovered in Lop + Urd treated groups, even though the rate of decrease varied (Fig. 4).

### Effect of Urd administration on regulation of the ability to secrete mucin

**Fig. 5:**
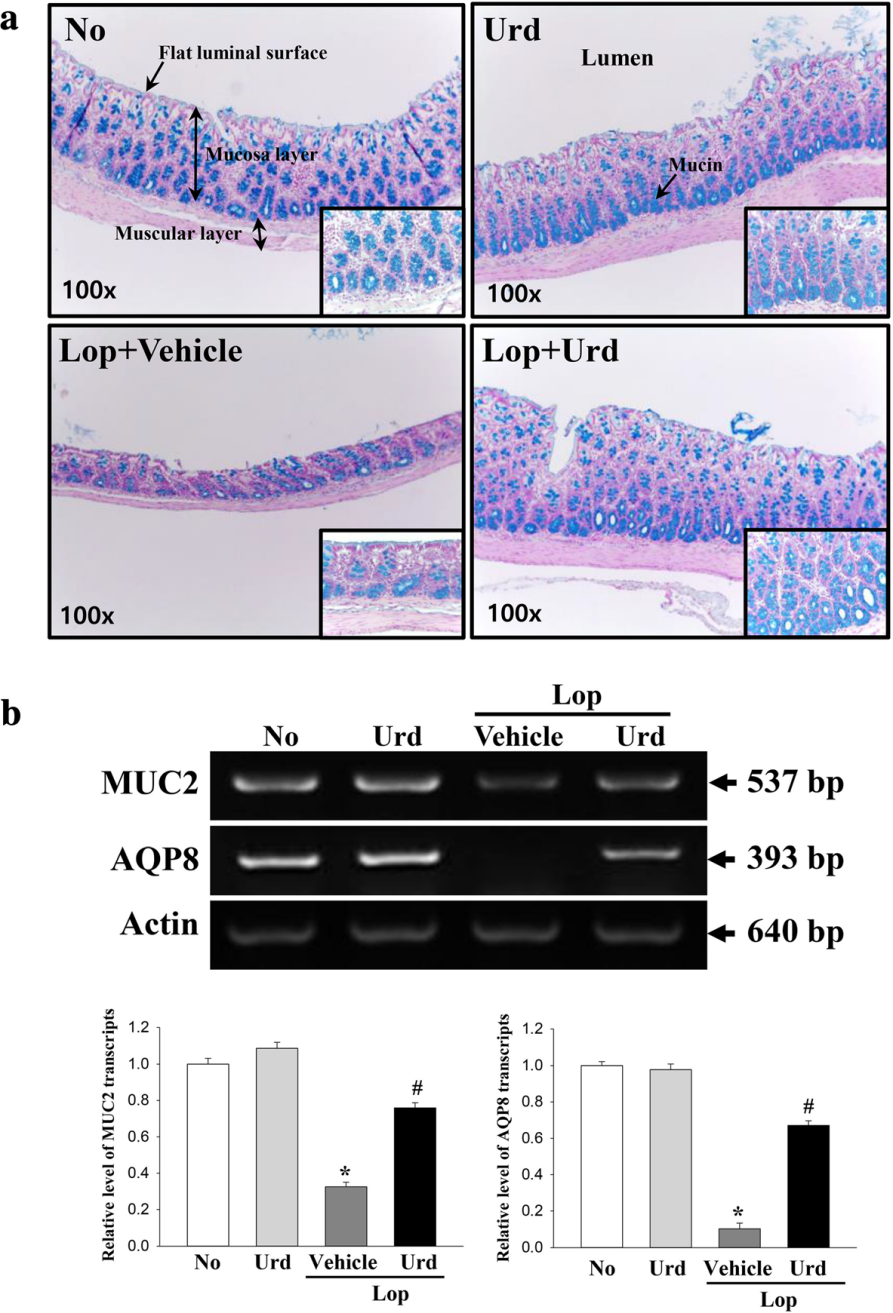
Detection of mucin secretion and membrane water channel expression. **a** Mucin secreted from crypt layer cells was stained with alcian blue at pH 2.5 and their images were observed at 100× magnification. The high magnification image (200×) is presented in the right corner of each figure. Five to six rats per group were assayed in triplicate by alcian blue staining. **b** The levels of MUC2 and AQP8 transcripts in the total mRNA of transverse colons were measured by RT-PCR using specific primers. After the intensity of each band was determined using an imaging densitometer, the relative levels of MUC2 and AQP8 mRNA were calculated based on the intensity of actin as an endogenous control. Five to six rats per group were assayed in triplicate by RT-PCR assays. Data represent the means ± SD of three replicates. *, *p* < 0.05 compared to the No treated group. ^#^, *p* < 0.05 compared to the Lop + Vehicle treated group

To determine if Urd treatment could enhance the ability to secrete mucin in the transverse colon, the levels of mucin were observed in tissue sections of the transverse colon stained with Alcian blue. In the No treated group and the Urd treated group, the region stained with dark blue indicating mucin was concentrated into the crypt in the mucosa layer of the transverse colon. The level of mucin was decreased in the Lop + Vehicle treated group and dramatically increased in the Lop + Urd treated group (Fig. 5a). A similar pattern was also detected in the expression level of MUC2 mRNA. The decrease in the level of this gene in the Lop + Vehicle treated group was significantly increased by 165% in the Lop + Urd treated group. Furthermore, we investigated whether mucin secretion induced by Urd treatment was accompanied by altered expression of a membrane water channel by measuring the level of AQP8 mRNA in the transverse colon of subset groups. The level of AQP8 mRNA was very similar to that of MUC2 mRNA. These values decreased by 90.5% in the Lop + Vehicle relative to the No treated group, but AQP8 mRNA was dramatically increased in the Lop + Urd treated groups (Fig. 5b).

### Confirmation of Urd effects in pRISMC

**Fig. 6:**
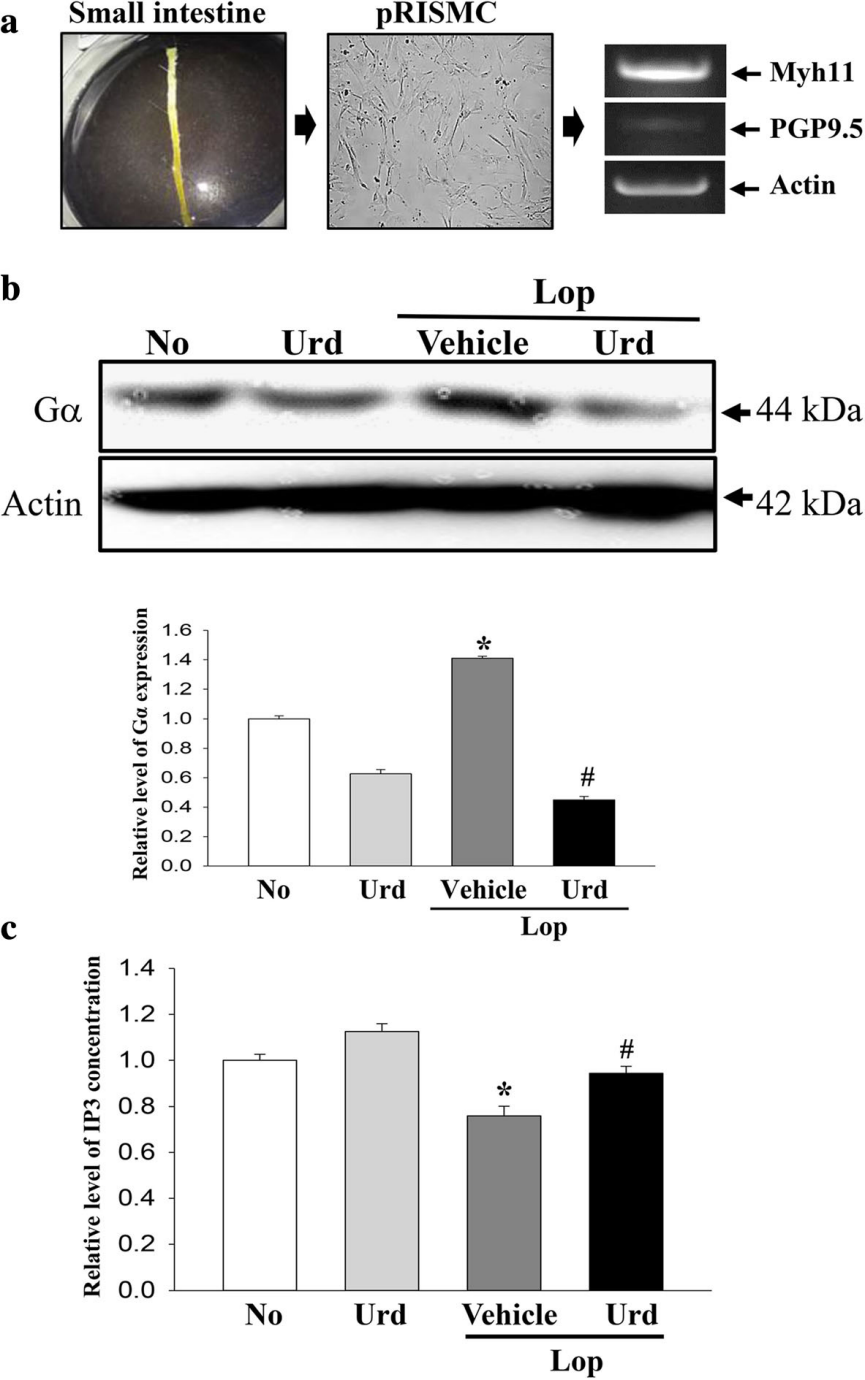
Detection of Gα expression and IP3 concentration in pRISMC. **a** pRISMCs were collected from the small intestines of infant rats and confirmed by RT-PCR analysis using specific primers. **b** Total cell lysate protein was extracted from Lop-pretreated pRISMC after treatment with the Urd. The levels of Gα expression were detected using specific antibodies. The actin level is also shown as an endogenous control. The band intensity of the three proteins was determined using an imaging densitometer and the relative level of each protein was calculated based on the intensity of actin protein as an endogenous control. **b** After treatment with Lop for 12 h, pRISMC were further incubated with Urd as described in the materials and methods. The IP3 concentration in the total cell lysate was measured using an ELISA kit that could detect IP3 at 5 pg/ml to 1000 pg/ml. Data represent the means ± SD of three replicates. *, *p* < 0.05 compared to the No treated group. ^#^, *p* < 0.05 compared to the Lop + Vehicle treated group

Finally, we confirmed the laxative effect of Urd in pRISMCs collected from the transverse colon. The expression of Gα protein was 42% higher in the Lop + Vehicle treated group than the No treated group. However, these levels were dramatically decreased after Lop + Urd treatment (Fig. 6b). Additionally, significant alterations were observed upon analysis of the IP3 concentration. The decrease in IP3 in the Lop + Vehicle treated group was significantly recovered after Lop + Urd treatment (Fig. 6c).

## Discussion

Herbal plants and natural products have recently received increased attention as novel therapeutic drugs for the treatment of constipation and its related diseases because they show a significant possibility for the prevention and treatment of inflammation related diseases [16, 19, 20]. Some studies have been focused on the identification of key compounds involved in constipation although they did not report key compounds with laxative effects. In an effort to identify drugs for the treatment of constipation, we investigated the therapeutic effects of Urd on Lop-induced constipated SD rats because Urd have been detected from extracts of several medicinal plants that have therapeutic effects against some human diseases. These included the aqueous extract of *Isatis indigotica*, which has anti-cancer and anti-viral effects [21, 22], and the extract of *Longan arillus*, which has anxiolytic-like effects [23]. In addition, Urd was detected in the alkaloid extract of *Pinelliae pedatisecta*, which has anti-cancer effects [24]. The results of the present study clearly demonstrated that Urd may induce laxative effects, including elevation of stool excretion and the recovery of histological changes induced by Lop injection in the transverse colon. Our data are the first to demonstrate that the laxative effects of Urd are tightly correlated with down-regulation of the mAChRs signaling pathway, up-regulation of the mucin secretion ability and water content capacity in the transverse colon.

Neurobiological roles of Urd have been investigated in many pharmacological studies, but none of these have provided direct evidence of a relationship between Urd and intestinal function [6]. Urd can bypass the blood brain barrier by binding one of two transporters, high affinity transporter (1–50 μM range) and low affinity transporter (100–800 μM range) [25, 26]. Urd also mediates as a novel neurotransmitter via purinergic receptor (P2 receptor) in response to extracellular purine and pyrimidines when it is not providing a substrate for phosphatidylcholine synthesis [27, 28]. It can also induce the creation of membrane and dendrites of neuronal cells through enhancement of phosphatidylcholine levels in the brain as well as neuronal differentiation and outgrowth via activation of NGF signaling [29, 30]. Furthermore, administration of some drugs containing uridine and uridine-5-monophosphate enhanced dopamine output from activated neurons by 11.6–20.5% and improved spatial short term memory, recognition, recall, attention and executive function [30]. Although the above studies suggested that Urd can be potentially used as laxative compounds, there are however, no direct scientific evidence on the therapeutic effect of Urd for chronic constipation. Therefore, the results of the present study provide the first evidence that the laxative effects of Urd may correlate with neuronal functions of the transverse colon. However, more studies are needed to elucidate the molecular mechanism responsible for the effects of Urd against chronic constipation.

Alterations in excretion factors such as number, weight and water contents of stools in Lop-injected rats were dramatically recovered by treatment with herbal medicines such as *Aloe ferox Mill*., *Liriope platyphylla* and *Mareya micrantha Mull*. [5, 15, 20]. However, the rate of increase varied in each group treated with different herbal medicine. The stool weight was enhanced by 176–272% in rats treated with the aqueous extract of *Mareya micrantha*, while it was increased by 71–143% in the case of *Aloe ferox Mill*. treatment [15, 20]. Moreover, an increase of 52–80% and 86% in the number of stools was measured in the group treated with the aqueous-methanol extract of *Fumaria parviflora* and aqueous extract of *L. platyphylla* (AEtLP) [5, 31]. In our study, the number, weight and water content of stools increased by 77.6, 92.3 and 110.9%, respectively, in the Lop + Urd treated group when compared with the Lop + Vehicle treated group. These findings were similar to those observed for in animals treated with several herbal medicines, although the level of each parameter extended in broad range.

Stool excretion, histopathological structure of colon, mucin secretion and mAChR expression are considered to be important factors for evaluation of constipation symptoms and therapeutic effects of some drugs [5, 15, 16, 32]. However, in the most studies conducted to date, the laxative effects were measured using stool excretion parameters, including number, weight, and water content, as well as histopathological features of the colon including thickness of the villus layer, crypt layer and muscle layer after the administration of various plant extracts [15, 20]. In this study, similar results were detected in the above parameters, although the alteration rate varied in each group. Therefore, these results indicate that Urd administration could improve Lop-induced constipation through stimulation of stool and urine excretion, recovery of the histopathological structure, and mucin secretion. OUr study also investigated the molecular mechanism responsible for the mAChR signaling pathway and mucin secretion in constipated SD rats treated with Urd. The results regarding the effects of Urd on the mAChR signaling pathway and mucin secretion provide basic information that will be useful to future studies of the causes of constipation and selection of targets for constipation treatment.

IP3 can also be considered one of the factors related to bowel movements because it induces increases in intracellular Ca^2+^ levels through a release from the sarcoplasmic reticulum [33]. Smooth muscle tone in the intestine is controlled by phosphorylation of the regulatory light chain (MLC) of myosin II. Ca^2+^/CaM-dependent myosin light chain kinase and myosin phosphatase regulated this process to control the velocity and force of actomyosin cross-bridging [34]. Therefore, we measured the IP3 level in pRISMC to investigate the effects of Urd response to Lop treatment. The results of the present study suggest that Urd may play an important role in pRISMCs collected from the small intestine of infant rats treated with Lop and that its effects are tightly correlated with those of laxatives that improve chronic constipation.

AQP, which is one of the membrane water channels that control water content in various cells, is connected to several diseases including congenital cataracts and nephrogenic diabetes insipidus [35]. Among several subtypes of AQPs distributed in the intestinal epithelial cells, AQP8 is primarily expressed in the absorptive epithelial cells and localized normally in colonic epithelial cells [36, 37]. The expression of AQP8 was observed as three major patterns based on their pathological condition. In most cases, the expression of this gene is downregulated in experimental colitis [38], inflammatory bowel diseases [38], diarrhea-predominant bowel syndrome (D-IBD) [39], melanosis coli [40], rotavirus-induced diarrhea [41] and ulcerative colitis [42]. However, AQP8 mRNA and protein levels were significantly enhanced in the colon of ulcerative colitis [43] and paraneoplastic normal tissues [44]. Furthermore, AQP8 was not expressed in the colonic mucosa of slow transit constipation rats treated with diphenoxylate solution [45]. In the present study, the level of AQP8 mRNA was significantly decreased in the Lop + Vehicle treated group when compared with the No treated group. This pattern was very similar to that of several bowel diseases, although the decrease rate varied. However, the level of AQP8 in the Lop treated group differed from that of the constipation induced by diphenoxylate solution. It is likely that the observed differences between studies can be attributed to the disease induction mechanism of the compound; however, more studies are needed to understand what other factors determine AQP8 expression.

Lop used in this study is widely applied to induce chronic constipation, including the extension of stool evacuation time and the delay of intestinal luminal transit SD rats and ICR mice through inhibition of water secretion [46] and smooth movement in the intestinal wall [47, 48]. However, this animal model induced by Lop injection could only simulate slow transit constipation among three afflictions categorized in human patients based on assessment of colonic transit and anorectal function; normal transit or irritable bowel syndrome, pelvic floor dysfunction (functional defecatory disorders) and slow transit constipation [49–51]. Therefore, the present study has some limits and restrictions to the clinical translation of results obtained from Lop induced rats to human conditions.

Alterations in endogenous metabolites were also examined in the serum of Lop-induced constipation rats. Among 35 endogenous metabolites, four amino acids (alanine, glutamate, glutamine and glycine) and six endogenous metabolites related to the glycolysis pathway (acetate, glucose, glycerol, lactate, succinate and taurine) were dramatically decreased after Lop treatment [52]. These results provide a possibility that Urd can rescue the Lop-induced constipation by interfering with the balance of metabolites because Urd plays a role in the glycolysis pathway of galactose [53]. However, additional studies are needed to determine if this is the case.

## Conclusions

The results of the present study provide the first strong evidence that Urd could induce the recovery of pathological symptoms including excretion parameters, histological structure, ultrastructure mucin secretion and water homeostasis in Lop-induced constipation SD rats. Our results also provide information that will help elucidate the laxative mechanism of Urd on the downstream signaling pathway and expression of mAChRs.

## Abbreviations

AQP8: Aquaporine 8
IP3: Inositol triphosphate
Lop: Loperamide
mAChR: Muscarinic acetylcholine receptors
mAChRs: Muscarinic acetylcholine receptors
PI-3K: Phosphoinositide 3-kinase
pRISMC: Primary smooth muscle of rat intestine cells
TEM: Transmission electron microscope
Urd: Uridine
